# Pelvic abscess after oocyte retrieval in women with endometriosis: A case series

**Published:** 2013-08

**Authors:** Bárbara Romero, Laura Aibar, Luis Martínez Navarro, Juan Fontes, Maria-Angeles Calderón, Juan Mozas

**Affiliations:** 1 ***Reproduction Unit, Universitary Hospital Virgen de las Nieves, Granada, Spain.***; 2 *Department of Obstetrics and Gynecology, Hospital of Santa Barbara, Puertollano (Ciudad Real), Spain.*

**Keywords:** *Oocyte retrieval*, *In vitro fertilization*, *Endometriosis*, *Pelvic inflammatorydisease*

## Abstract

**Background:** Pelvic inflammatory disease with progression to pelvic abscess is a rare complication after oocyte retrieval during in vitro fertilization cycles. However, in patients with endometriosis the risk appears to be increased. Many authors agree on the need for antibiotic prophylaxis during the oocyte retrieval in these patients, but there is no consensus regarding the best antibiotic.

**Case:** We discuss 3 clinical cases of tubo-ovarian abscess in women with endometriosis after oocyte retrieval despite antibiotic prophylaxis between 2004 and 2011 at our center, and discuss our experience in the context of earlier reports.

**Conclusion:** It is unclear whether antibiotic prophylaxis is necessary in these women, and which antibiotic is best. Only douching with povidone-iodine appears to decrease the rate of pelvic infection.

## Introduction

Endometriosis occurs in 25% to 50% of women with infertility (1, 2). In vitro fertilization is an effective treatment for these women, and although pelvic inflammatory disease (PID) is an infrequent complication of oocyte retrieval (OR) (0.3-0.4%), endometriosis is a risk factor to date only 9 cases of abscess after OR have been reported (3-15). We describe 3 cases of pelvic abscess after OR in women with endometriosis in southern Spain between 2004 and 2011.

## Case series

**Case 1**

A 29 year old woman with a history of right ovarian cystectomy for endometriosis and severe adhesion syndrome was given 1500 mg cefuroxime intravenously during OR. Two bilateral endometriomas were seen and were not punctured. She became pregnant but later miscarried. During the second cycle cefuroxime was again used for prophylaxis during OR and the endometriomas were not punctured. She did not get pregnant. 

One month later she was hospitalized for an 8 cm pelvic abscess that required surgical drainage after her clinical status had improved with intravenous antibiotics.

**Case 2**

**Figure 1:**
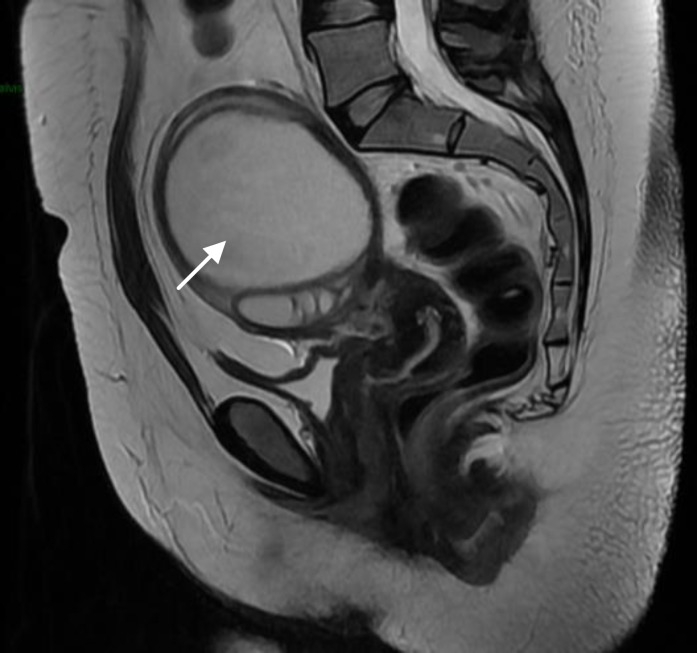
Pelvic abscess in a 32 years old women (Case 2).

A 32 year old woman with a 3 cm endometrioma diagnosed by ultrasound in her right ovary received vaginal douching during OR with povidone-iodine and saline solution. In addition, 1 g azithromycin was given orally and 1 g ceftriaxone was given intravenously in a single doses. Her endometrioma was punctured during OR. She did not get pregnant. Two months later she was hospitalized for a 9 cm pelvic abscess that did not resolve with intravenous antibiotics and required surgical drainage (Figure 1). 

**Case 3**

**Figure 2:**
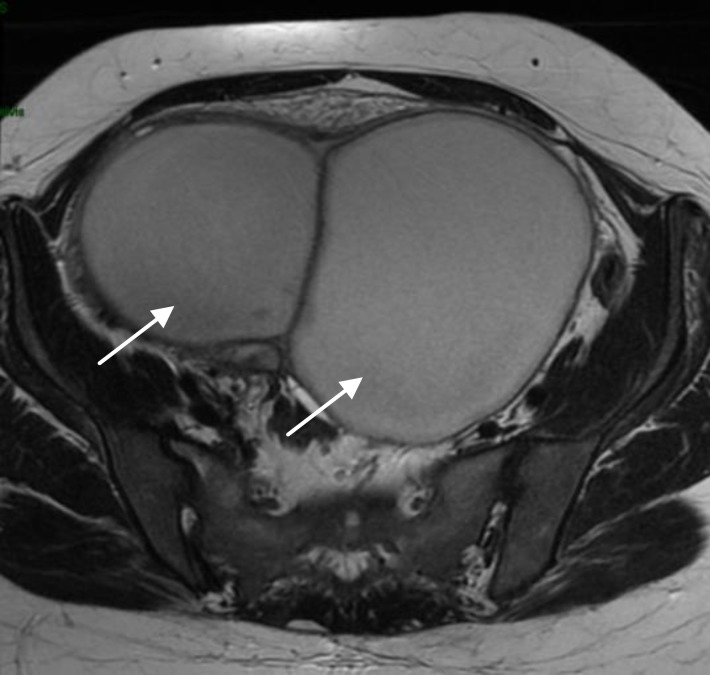
Pelvic abscess in a 37 years old women (Case 3).

A 37 year old woman with a history of right ovarian cystectomy for endometriosis received vaginal douching during OR with povidone-iodine and saline solution; in addition, 1 g azithromycin was given orally. A 4 cm endometrioma was seen and was not punctured. She did not conceive. Three weeks later she was hospitalized for a 9 cm pelvic abscess (Figure 2), and responded favorably to intravenous antibiotic treatment. However, her abscess required surgical drainage and necessitated right adnexectomy.

## Discussion

The frequency of PID after OR is difficult to establish since it is a rare complication. At our center, of 4319 OR procedures done between 2004 and 2011, there were only 3 cases of pelvic abscess (0.07%), all in women with endometriosis. One of the limitations that may contribute to this low complication rate is the lack of a pathological diagnosis of endometriosis, although the sensitivity and specificity of the diagnosis of endometrioma with ultrasonography were shown to be high (16). In two of our patients the diagnosis was confirmed pathologically, but in the third patient the diagnosis was based on ultrasound findings only. 

Moini *et al* identified 10 cases of PID among 5958 OR procedures, and 8 of these women had endometriosis (7). Chen *et al* analysed the association between endometriosis and pelvic abscesses in 3215 women and concluded that old blood in the endometrioma was an excellent culture medium; accordingly, they considered endometriosis to be an important risk factor for PID (10).

Surgical treatment of endometriomas reduces the risk at the expense of diminishing follicular reserve, and thus prolonging stimulation cycles and increasing the cost of in vitro fertilization (17-20). Both the Royal College of Obstetricians and Gynecologists and the European Society of Human Reproduction and Embryology recommend resection for endometriomas measuring 4 cm or larger in order to reduce the risk of infection, among other aims (21, 22). 

Although there is no evidence of the efficacy of antibiotic prophylaxis during OR, most authors have used prophylaxis for women with endometriosis (23). Benaglia *et al* attempted to determine the frequency of pelvic abscesses in women after OR who were treated prophylactically with ceftriaxone 1 g intramuscularly for 4 days (24). Among 214 OR procedures, the involved ovary was not punctured in 12% of the cases, and in 3% the endometrioma was punctured accidentally. There were no cases of abscess. 

Egbase *et al* used ceftriaxone 2 g+ metronidazole 1 g intravenously, and among cultures of the embryo transfer catheter, 78% were negative, versus 30.9% in women who were not given prophylactic antibiotics (25). The conception rate was lower in women with a positive culture (18.7% vs. 41.3%). Weinreb *et al *treated oocyte donors prophylactically with cefoxitin 2 g or clindamycin 900 mg intravenously, and found that this reduced the risk of PID by 0.4% to 0% (26). However, the use of cefazolin for prophylaxis by Younis *et al* did not prevent the appearance of PID (9). 

To our knowledge only 9 cases of pelvic abscess after OR in women with endometriosis have been reported in 7 different studies (7, 9, 11-15). Eight of these women had received antibiotic prophylaxis, and in 6 of them the endometrioma had been punctured. These numbers suggest that antibiotic prophylaxis is not effective in preventing PID, although it would be interesting to know how many cases have been prevented by prophylaxis. Pelvic abscesses after OR may be underreported, as the present case series appears to suggest. 

At our centre all women with endometriosis who undergo in vitro fertilization are given antibiotics prophylactically during OR with azithromycin 1 g orally or ceftriaxone 1 g intramuscularly, or less frequently with cefuroxime 1500 mg or cefazolin 2 g intravenously. Yet despite antibiotic prophylaxis, 3 of the women at our centre developed pelvic abscesses (which required hospital treatment), and one woman, as in the study by Younis *et al* was resistant to antibiotic treatment (9). The endometrioma was punctured in only 1 of the 3 women described here, and 2 of them had a history of ovarian surgery- an antecedent that Chen *et al* identified as an additional risk factor (10).

Tsai *et al* showed that vaginal douching with povidone-iodine before OR decreased the risk of o PID, although Van Os *et al* reported a lower conception rate in these women (8, 27). Hannoun *et al* have suggested the use of povidone-iodine followed by saline solution (28). Other preventive measures are the use of strict asepsis in the surgical field, avoiding successive punctures of the vaginal wall and ovarian capsule, and avoiding puncture and aspiration of the endometrioma during OR (23, 24). 

## Conclusion

Endometriomas 4 cm in diameter or larger should be respected. It is unclear whether antibiotic prophylaxis should be used during OR, or which agents are the most effective. Vaginal douching with povidone-iodine followed by saline solution appears to reduce the risk of PID. 
